# Pediatric Motor Inflammatory Neuropathy: The Role of Antiphospholipid Antibodies

**DOI:** 10.3390/brainsci10030156

**Published:** 2020-03-07

**Authors:** Claudia Brogna, Marco Luigetti, Giulia Norcia, Roberta Scalise, Gloria Ferrantini, Beatrice Berti, Domenico M. Romeo, Raffaele Manna, Eugenio Mercuri, Marika Pane

**Affiliations:** 1 Pediatric Neurology and Nemo Clinical Centre, Fondazione Policlinico Universitario A. Gemelli IRCSS, Università Cattolica del Sacro Cuore, 00168 Roma, Italy; giulanorcia@yahoo.it (G.N.); robscal@hotmail.it (R.S.); ferrantinigloria@yahoo.it (G.F.); beatriceberti@gmail.com (B.B.); eugeniomercuri@unicatt.it (E.M.); marika.pane@policlinicogemelli.it (M.P.); 2 Pediatric Neuropsichiatric Unit, ASL, 83100 Avellino, Italy; 3 Institute of Neurology, Fondazione Policlinico Universitario A. Gemelli IRCSS, Università Cattolica del sacro Cuore, 00168 Roma, Italy; mluigetti@gmail.com; 4 Pediatric Neurology, Fondazione Policlinico Universitario A. Gemelli IRCSS, Università Cattolica del Sacro Cuore, 00168 Roma, Italy; domenicomarco.romeo@policlinicogemelli.it; 5 Periodic Fevers Research Centre and rare disease. Department of Internal Medicine, Fondazione Policlinico Universitario A. Gemelli IRCSS, Università Cattolica del Sacro Cuore, 00168 Roma, Italy; raffaele.manna@unicatt.it

**Keywords:** antiphospholipid antibodies, pediatric motor neuropathy, IVIG

## Abstract

We report the clinical case of a nine-year-old girl who presented with progressive motor neuropathy, revealed via the detection of a higher delay in F-wave recording using digitalized nerve conduction/electromyography. Since the lupus anticoagulant (LAC) positivity, detected using diluted Russell viper venom time (dRVVT), switched to persistent serological anticardiolipin immunoglobulin G (IgG) positivity, a possible non-thrombotic antiphospholipid antibody (aPL)-related clinical manifestation was suspected, and intravenous immunoglobulin treatment (IVIG) was started. The IVIG treatment was well tolerated and the complete resolution of motor impairment was obtained after the third IVIG infusion. Our findings suggest that it could be useful to check for antiphospholipid antibodies in children with a rapid onset of progressive neurological signs in order to provide the beneficial use of IVIG in the treatment of pediatric aPL neurological conditions.

## 1. Introduction 

In the last few years, an increased number of children affected by underlying autoimmune diseases associated with neurological manifestations as the first clinical sign was evidenced [1]. In most cases, the timing and the type of immunomodulatory therapy could be essential in allowing a successfully outcome. Therefore, an early and detailed clinical evaluation, together with accurate patient medical history, represents a step in diagnosis, and they could help clinicians to address a more specific early type of treatment. In most cases, nerve conduction studies (NCS) and electromyography (EMG) could support the clinical diagnosis, confirming a more immune than degenerative or hereditary pattern, even in childhood [2]. We report a pediatric clinical case characterized by a progressive inflammatory motor polyneuropathy successfully treated with intravenous immunoglobulin (IVIG), in the presence of transient lupus anticoagulant (LAC) positivity later switching to persistent anticardiolipin immunoglobulin G (IgG) positivity. 

## 2. Case Presentation

A healthy nine-year-old child of Italian parents progressively presented with gait disturbance, frequent falls, and difficulty jumping and rising from the ground, associated with fatigability. She was born at term after an uncomplicated pregnancy, and psychomotor milestones were reported as normal. There was no familiar history for neurological disease or hereditary and degenerative neuropathy. The parents did not report any signs or history of infections before the onset of the neurological symptoms, and the girl did not show other neurological signs like fasciculations or cramps. When she was admitted to our unit seven months after the onset of the disease, the neurological examination showed a mild muscular weakness (lower limbs more than upper limbs, with major involvement of proximal muscles with respect to distal muscles), absent tendon reflexes at the lower limbs, and difficulty walking on heels and jumping, as well as unstable static balance and impaired dynamic balance. Her height and weight were 147 cm (97th percentile on growth chart) and 35 kg (75th–90th percentile on growth chart). Some functional motor assessments, including the Six-Minute Walking test (6MWT) [3], the North Star assessment [4], and the Hammersmith Functional Motor Scale (HFMS) [5], showed diffuse impairment with evidence of fatigue and a more proximal than distal weakness. The girl was able to arise from the ground, showing incomplete Gowers signs and the ability to run 10 m without double stance in 8.70 s. Spinal cord magnetic resonance with contrast was normal, and a previous genetic test excluded Survival Motor Neuron gene 1 (SMN 1) deletion compatible with a motoneuron disease. Several laboratory assessments were performed excluding any evidence of acute inflammatory infection, including viral infections. Moreover, paraneoplastic syndrome or any autoimmune disease, hereditary clotting exams, and coagulation screening, including activated partial thromboplastin time (aPTT), were also investigated and were found in the normal range (Table 1). Antiphospholipid antibodies (aPL) including lupus anticoagulant (LAC), anti-cardiolipin antibodies (IgG and IgM), and anti-β2-glycoprotein I antibodies (IgG and IgM) were also investigated at the admission as part of the autoimmune assessment. Initially, only LAC positivity was found, detected using the diluted Russell viper venom time (dRVVT) assay (coagulation time of dRVVT 38.9 s confirmed by dRVVT ratio 1.35). The lymphocyte profile evidenced an increased CD4/CD8 ratio of 2.17 (1.10–1.40). Electromyographic examination showed mild denervation signs (positive sharp waves and fibrillation potentials) with rapidly firing motor unit potentials of normal amplitude in both lower limb muscles (right rectus femoris and tibialis anterior). Nerve conduction studies were unremarkable in both lower and upper limbs. However, F-wave mean latency was markedly prolonged (considering height according to the patient’s age), stimulating both the tibial nerve (52 ms) and the median nerve (29 ms) of both limbs. Neurophysiological findings were suggestive of chronic inflammatory demyelinating polyneuropathy (CIDP). Therefore, intravenous immunoglobulin (IVIG) treatment was started at a dose of 0.4 mg/kg/day for five days. After the first month of treatment, the girl showed a rapid improvement in motor performance and a reduction in proximal weakness. She did not report pharmacological side-effects. The girl progressively regained the ability to walk without evidence of fatigue, and she was able to run with a double stance and arise from the ground without any Gowers signs; she also obtained a progressively more stable static and dynamic balance (Video S1). The girl also reported an increased improvement in both 6MWT and HFMN functional motor tests (Table 2). The aPL therapy was repeated every 12 weeks according to the laboratory criteria for antiphospholipid syndrome (APS). LAC was found to be positive after 12 weeks, i.e., the first detection, before starting the third immunomodulatory therapy (coagulation time of dRVVT 44.5 s confirmed by dRVVT ratio 1.37), and it was found to be negative at the sixth month of IVIG treatment, when the first positivity of anticardiolipin, detected by chemiluminescence immunoassay, started to appear (25.0 U/mL), persisting six months later (24.2 U/m:). In the following months, after IVIG, the lymphocyte profile showed a progressive reduction in the CD4/CD8 ratio (1.94 ratio one month later, 1.87 ratio two months later, 1.81 ratio three months later). The other features related to APS, both classical (mainly thrombosis, whether arterial or venous) and non-classical (livedo reticularis, memory loss, thrombocytopenia), were not present, except for sporadic episodes of migraine and some concentration difficulties reported by the parents during the first months. 

IVIG treatment was administered every month for five months, and then the girl passed to a monthly subcutaneous immunoglobulin treatment with a good outcome. One year later, the IVIG treatment F-wave mean latency was in the normal range. Her height and weight were 152 cm (97th percentile on growth chart) and 42 kg (75th–90th percentile on growth chart).

## 3. Results and Discussions

Inflammatory neuropathies are a heterogeneous group of autoimmune disorders affecting the peripheral nervous system, which could cause significant and often permanent disability in many affected patients. Although, in the last few years, the understanding of disease mechanisms in inflammatory neuropathies was improved, several important aspects remain poorly understood and remain to be experimentally addressed. Chronic inflammatory demyelinating polyradiculoneuropathy (CIDP) is the most common chronic inflammatory neuropathy in children, and it is usually characterized by slowly progressive, symmetric, proximal, and distal paresis and sensory dysfunction. This young girl developed a form of CIDP in the context of transient LAC and persistent (albeit at low titers) IgG anticardiolipin antibodies. The association between anticardiolipin and CIDP is not yet well elucidated. Only few CIDP adult patients were found to be positive for anticardiolipin antibodies (aCL), but this result was reported as not significant, not APS related, and probably associated with myelin damage or cross-reactions with other anti-myelin antibodies [6]. 

In the last few years, APS was increasingly recognized, both as a secondary condition to other autoimmune diseases, mainly systemic lupus erythematosus, and as a primary condition [1,7,8,9,10]. APS and aPL are also increasingly being recognized in children. More specifically, the prevalence of transient non-pathogenic aPLs like anticardiolipin antibodies was reported to range from 2% to 82% in healthy children, frequently related to childhood infections, while thrombotic events are rarely reported in children with true aPL [11]. 

However, it is known that, most often, non-thrombotic clinical signs, mainly in children, could precede the onset of APS, before the occurrence of thrombosis, which was also reported in the Ped-APS Registry [7]. The mechanism via which aPL could act is becoming of increasing interest. It is known that aPL can produce neurological signs indirectly causing brain endothelial dysfunction or directly damaging the cerebral white matter and structures binding to the neuronal cell surface of the basal ganglia or other neurological structures responsible for neurological impairment [8,9]. Despite progressive emerging evidence of a direct interaction between aPL and neural tissue, recent diagnostic criteria for APS do not consider neuropsychiatric manifestations as a valid criterion, while also considering that neurological manifestations are rare, even in the Ped-APS Registry.

In our opinion, the presence of aPL positivity associated with neuropathy and the exclusion of all other relevant causes of acquired or genetic neuropathy could be considered as a risk factor for developing a typical subgroup of CIDP, or it could represent a possible non-thrombotic clinical aPL-related manifestation. For these reasons, it could be important to follow the clinical evolution of children with the same neurological history. 

Furthermore, more recently, only few studies reported evidence of the beneficial use of IVIG to treat pediatric aPL-related chorea [9,10,12,13,14,15]. Our clinical case confirmed the importance of the early use of IVIG treatment in CIDP children with aPL positivity. IVIG should be considered as a first-line and gold-standard treatment in children with neurological immune-related conditions. Moreover, in aPL-related conditions, IVIG could be useful not only for the clinical outcome but also to prevent possible prothrombotic injury [16]. 

This case report confirmed the importance of an accurate medical history to address a more specific investigational exam, such as neurophysiological examination, which could help to perform early diagnosis and IVIG treatment.

## 4. Conclusions

In conclusion, our findings suggest checking aPL in children with a rapid onset of progressive neurological signs in order to provide the beneficial use of IVIG. However, since no diagnostic criteria nor a diagnostic test to firmly attribute or discard the plausible role of aPL exist, a determination of such antibodies could be considered as part of the diagnostic procedure in these cases. 

Therefore, it is possible to consider aPL antibodies as a marker underpinning a more specific immune mechanism. However, it is necessary to collect other case reports and prospective studies on children to allow a better evaluation in a large series and to better comprehend the mechanism of efficacy of IVIG in neurological aPL manifestations. 

## Figures and Tables

**Table 1:** Laboratory assessment before intravenous immunoglobulin treatment (IVIG) treatment.

**Autoimmunity**	ASMA, AMA, ENA, anti-nucleosome, anti-centromere, anti-Jo1, p ANCA/c ANCA, anti-dsDNA, anti-SCL 70, anti-citrulline, anti-endomysial and anti-transglutaminase, anti-GAD, cryoglobulins, immune complexes, C3, C4, anti-gangloside (GM1, GM2, GM3, GD1a, GD1b, gt1B, gq1B, AB2 IgG), anti-aquaporine 4 antibodies	All Neg
**aPL**	Lupus anticoagulant,	LAC +, drVV ratio +
	Anti β2 glycoprotein antibodies	Neg
	Anti-cardiolipin antibodies	Neg
	Anti-prothrombin antibodies	Neg
**Onco-neural antibodies**	Anti Yo, anti-Ri, anti-Hu, anti-AGNA, anti-Me	Neg
**Serum exams**	Erythrocyte sedimentation rate, C-reactive protein, ASO titer, ammonium, serum lactate, serum ceruloplasmin, copper serum and urine levels, rheumatoid factor, serum free thyroid hormones	All Neg
**Anti-neuron antibodies**	Anti-NMDAR, anti-GABA b, anti-Lgi-1, anti-CASPR 2	All Neg
**Serology**	*TORCH complex*	Neg IgM, IgG
	*Enterovirus*	Neg IgM, IgG
	*Parvovirur B 19*	Neg IgM, IgG
	*Epstein Barr virus*	Neg IgM VCA, + IgG VCA
	*Mycoplasma virus*	Neg IgM, + IgG
	*Varicella virus*	Neg IgM, + IgG
	*Measles*	Neg IgM, + IgG
	*Adenovirus*	Neg IgM, IgG
	*Mumps*	Neg IgM, + IgG
	*Echovirus*	Neg IgM, IgG
	*A Influence,*	Neg IgM, IgG
	*B Influence*	Neg IgM, + IgG
	*Coxsackie virus*	Neg IgM, IgG
	*Borrelia burgdorferi*	Neg IgM, IgG
	*Parvovirus B19*	Neg IgM, IgG
**Hereditary clotting exams**	Antithrombin, protein C and protein S, factor V Leiden, prothrombin 20210A gene mutation, polymorphism of methylene tetrahydrofolate reductase	All Neg

ASMA: anti-smooth muscle antibody, AMA: anti-mitochondrial antibodies, ENA: anti-extractable nuclear antigens antibody, anti-Jo 1: anti-histidyl transfer RNA (tRNA) synthetase, p ANCA-C ANCA: anti-neutrophil cytoplasmic antibodies, dsDNA: double-stranded DNA, anti-SCL70: anti-topoisomerase I antibodies, anti-GAD: antibodies to glutamic acid decarboxylase; anti-Yo: Purkinje cell cytoplasmic antibodies type 1, anti-Ri: type II anti-neuronal nuclear antibodies (ANNA-2), anti-Hu: type 1 anti-neuron-specific cell nuclear antibodies (ANNA-1), anti-AGNA: anti-glial nuclear antibodies, anti-Me: anti-matrix metallopeptidase 12 antibodies, anti-NMDAR: *N*-methyl-d-aspartate receptor antibodies, anti-GABA b: γ-aminobutyric acid-B receptor antibodies, anti-Lgi-1: leucine-rich glioma-inactivated protein 1 antibodies, anti-CASPR 2: contactin-associated protein-like 2 antibodies, TORCH: toxoplasmosis, rubella, cytomegalovirus, and herpes simplex virus; Neg: negative.

**Table 2:** Motor functional assessments before and after IVIG.

IVIG Treatment	HFMS	North Star		6MWT
		10 m Item	Rise from Floor Item	
Before IVIG	56/66 (squat difficulty)	8.70 s no double stance	9.38 s, incomplete Gowers sign	323 m
1 Month after IVIG	64/66 (mild diff squat)	5.65 s no double stance	2.21 s, high kneeling	489 m
2 months after IVIG	65/66 (incomplete squat)	5.52 s with double stance	5.52 s, high kneeling	517 m
4 months after IVIG	66/66 (complete squat)	3.23 s with double stance	2.48 s, high kneeling	571 m
12 months after IVIG	66/66 (complete squat)	2.54 s with double stance	2.34 s, high kneeling	581 m

IVIG: intravenous immunoglobulin treatment, 6MWT: Six-Minute Walking test, HFMS: Hammersmith Functional Motor Scale.

## Supplementary Materials

The following are available online at https://www.mdpi.com/2076-3425/10/3/156/s1, Video S1: Functional motor outcome before and after IVIG treatment.
